# ALKBH10B, an mRNA m^6^A Demethylase, Modulates ABA Response During Seed Germination in Arabidopsis

**DOI:** 10.3389/fpls.2021.712713

**Published:** 2021-07-27

**Authors:** Jun Tang, Junbo Yang, Hongchao Duan, Guifang Jia

**Affiliations:** Beijing National Laboratory for Molecular Sciences, Key Laboratory of Bioorganic Chemistry and Molecular Engineering of Ministry of Education, Synthetic and Functional Biomolecules Center, College of Chemistry and Molecular Engineering, Peking University, Beijing, China

**Keywords:** Arabidopsis, *N*^6^-methyladenine, ALKBH10B, seed germination, ABA, osmotic stress

## Abstract

As the most abundant and reversible chemical modification in eukaryotic mRNA, the epitranscriptomic mark *N*^6^-methyladenine (m^6^A) regulates plant development and stress response. We have previously characterized that ALKBH10B is an Arabidopsis mRNA m^6^A demethylase and regulates floral transition. However, it is unclear whether ALKBH10B plays a role in abiotic stress response. Here, we found that the expression of *ALKBH10B* is increased in response to abscisic acid (ABA), osmotic, and salt stress. The *alkbh10b* mutants showed hypersensitive to ABA, osmotic, and salt stress during seed germination. Transcriptome analysis revealed that the expression of several ABA response genes is upregulated in *alkbh10b-1* than that of wild type, indicating ALKBH10B negatively affects the ABA signaling. Furthermore, m^6^A sequencing showed that ABA signaling genes, including *PYR1*, *PYL7*, *PYL9*, *ABI1*, and *SnRK2.2* are m^6^A hypermethylated in *alkbh10b-1* after ABA treatment. Taken together, our work demonstrated that ALKBH10B negatively modulates ABA response during seed germination in Arabidopsis.

## Introduction

*N*^6^-methyladenine (m^6^A) is the most abundant chemical modification in eukaryotic mRNA, and it is dynamically reversible (Jia et al., 2011; Zheng et al., 2013). Different protein complexes (“writer,” “eraser,” and “reader”) can “write,” “erase,” and “recognize” the m^6^A modification in mRNA. In mammals, the “writer” complex of RNA m^6^A mainly consists of three core subunits: METTL3 (methyltransferase like 3), METTL14, and WTAP (Wilms’ tumor 1-associating protein) (Liu et al., 2014; Ping et al., 2014). In addition, the m^6^A modification in RNA can be erased by its “eraser,” such as FTO (fat mass and obesity-associated protein) and ALKBH5 (alkylated DNA repair protein AlkB homolog 5) (Jia et al., 2011; Zheng et al., 2013). As an epitranscriptomic mark, m^6^A modification through its “reader,” such as the YTH family proteins, regulates RNA processing and metabolism, including alternative splicing, mRNA nuclear export, RNA stability maintenance, translation regulation, and pri-miRNA processing (Fustin et al., 2013; Wang et al., 2014, 2015; Alarcon et al., 2015; Meyer et al., 2015; Xiao et al., 2016).

RNA m^6^A modification is a conservative chemical modification. The mechanisms of “writing,” “erasing,” and “recognizing” of m^6^A in plants are similar to mammals. In Arabidopsis, the core subunits of RNA m^6^A methyltransferase complex includes MTA (homolog of human METTL3), MTB (homolog of human METTL14), and FIP37 (homolog of human WTAP). The core subunits of RNA m^6^A methyltransferase are essential proteins for embryonic development (Zhong et al., 2008; Shen et al., 2016; Ruzicka et al., 2017). In rice, the m^6^A methyltransferase are also essential for rice growth and development; mutation of OsFIP causes microspore degradation and affects its fertility (Zhang et al., 2019). There are 13 YTH domain proteins in Arabidopsis (Zhou et al., 2018). ECT2/ECT3/ECT4 and CPSF30-L have been confirmed as Arabidopsis m^6^A reader proteins. ECT2 promotes stability of m^6^A-modified mRNA and regulates trichome branching (Wei et al., 2018). ECT2/ECT3/ECT4 redundantly regulate the development of Arabidopsis epidermis (Arribas-Hernandez et al., 2018; Scutenaire et al., 2018; Wei et al., 2018). CPSF30-L recognizes m^6^A-modified FUE signal to mediate alternative polyadenylation and regulate flowering time, ABA response, and nitrogen metabolism (Hou et al., 2021; Song et al., 2021). The demethylases fine tune RNA m^6^A modification level, thus affect the dynamic state and reversibility of m^6^A. ALKBH9B and ALKBH10B are Arabidopsis RNA m^6^A demethylase and play important roles in regulating floral transition and viral infection of alfalfa mosaic virus (AMV). ALKBH10B mediates m^6^A demethylation in *SPL3*, *SPL9*, and *FT* transcripts and regulates their mRNA expression levels through m^6^A-mediated mRNA degradation pathway, thereby affects flowering time (Duan et al., 2017). ALKBH9B is driven to demethylate m^6^A in viral RNA of AMV through the specific interaction with the coat protein of AMV and positively regulates AMV accumulation and systemic invasion (Martinez-Perez et al., 2017). SIALKBH2 is identified as tomato m^6^A demethylase and regulates the ripening of tomato fruit. SIALKBH2 regulates the m^6^A level in the mRNA of DNA 5mC demethylase SIDML2 and affects its expression through m^6^A-mediated mRNA decay (Zhou et al., 2019).

Seed germination is the beginning of a new life cycle in higher plants, which is regulated by internal and environmental signals. The environmental signals perceived by seeds can be incorporated into endogenous hormonal signals. Abscisic acid (ABA) and gibberellic acid (GA) are regarded as the main hormones regulating seed germination (Seo et al., 2006; Shu et al., 2016). ABA positively regulates seed dormancy and inhibits seed germination, while GA acts antagonistically to release dormancy and to initiate seed germination (Shu et al., 2013). In this study, we found ABA, osmotic, and salt stress increase the expression of m^6^A demethylase *ALKBH10B*, which depends on ABI1. Further studies revealed that ALKBH10B plays a role in seed germination by negatively affecting ABA response genes. Moreover, we also found several ABA signaling genes with hypermethylated m^6^A peaks in the *alkbh10b* mutant, including *PYR1*, *ABI1*, *SnRK2.2*, *PYL7*, and *PYL9*. In all, this study revealed a negative regulatory role of ALKBH10B in ABA response during seed germination.

## Materials and Methods

### Plant Materials and Growth Conditions

*Arabidopsis thaliana* materials used in this study: *alkbh10b-1* (Salk_004215), *alkbh10b-2* (Salk_107289), and *ALKBH10Bp:ALKBH10B/alkbh10b-1* were derived from previous study, all in the Columbia-0 ecotype (Col-0) background (Duan et al., 2017); *abi1-1* (CS22) were in the Landsberg erecta (Ler) background.

All plants were grown at 22°C under long-day conditions (16 h light/8 h dark, white fluorescent tubes, 150–200 μmol m^–2^ s^–1^), and mature seeds of each genotype were collected at the same day, seeds were dried and stored at room temperature. For the seed germination assay, seeds were surfaced sterilized with 20% bleach for 10 min, then rinsed five times with sterile water, and plated on half strength Murashige and Skoog (MS) medium containing 0.8% sucrose, supplemented with various concentrations of ABA (0, 0.5, and 1 μM), 300 mM mannitol, or 150 mM NaCl, respectively. Plates were kept at 4°C in the dark for 3 days, and transferred to a culture room at 22°C under long-day conditions. Radicle emergence was scored after the indicated time intervals.

### Quantitative Reverse Transcription-PCR Analysis

Seven-day-old seedlings of Col-0 were used to evaluate *ALKBH10B* expression under external signals treatment. Seven-day-old seedlings were transferred to solid 1/2 MS medium, medium with 300 mM mannitol, 150 mM NaCl, or liquid 1/2 MS medium, medium with 50 μM ABA or 50 μM MeJA, respectively. Seedlings were collected after treatment for different times. Total RNA was isolated with Trizol reagent, and 1 μg total RNA was used for reverse transcription by PrimeScript RT reagent kit with gDNA Eraser (Takara). qRT-PCR was performed using SYBR Green Master Mix (YEASEN) on a ViiA 7 Dx (Applied Biosystems). All qRT-PCRs were performed in triplicate using total RNA samples extracted from three independent biological replicates. The 2^–ΔΔCT^ method was used to calculate the gene expression levels. *At2G28390* was used as an internal control (Czechowski et al., 2005). All primers used are listed in Supplementary Table 2.

### m^6^A Sequencing

Seven-day-old seedlings of *alkbh10b-1* and Col-0 were treated with half strength liquid MS medium supplemented with 50 μM ABA for 3 h, and the total RNA were extracted. Poly (A)^+^ RNA was isolated from total RNA using oligo(dT)_25_ Dynabeads (Thermo Fisher Scientific). 5 μg poly(A)^+^ RNA was fragmented into 100–150 nt by Magnesium RNA fragmentation module (NEB). m^6^A immunoprecipitation and library preparation were performed using the EpiMark *N*^6^-Methyladenosine enrichment kit (NEB). Input and RNA eluted from m^6^A-IP were used to prepare libraries with NEBNext Ultra II RNA Library Prep Kit. Sequencing was performed on an Illumina HiSeq X Ten machine in pair-end mode with 150 bp per read (Genewiz).

### Analysis of m^6^A-Seq Data

Sequence data were analyzed according to the procedure described by Song et al. (2021). Briefly, adapters and low-quality reads were trimmed by Cutadapt (v1.18) (Martin, 2011), clean reads were mapped to TAIR10 by HISAT2 (v2.1.0) (Pertea et al., 2016). PCR duplication were filtered by Picard Toolkit. The m^6^A peaks were identified using the MACS2 (Zhang et al., 2008) peak-calling algorithm based on enrichment criteria (IP/Input) ≥2 and FDR <0.05. Hypermethylated m^6^A sites were identified by MeTDiff (Cui et al., 2018) based on enrichment criteria fold change ≥2 and FDR <0.05. To evaluated the m^6^A distribution in transcripts, we divided TAIR10 transcripts into five-non-overlapping regions: 5′UTRs, Start_Codon (100-nucleotide window centered on the start codon), coding sequences (CDSs), Stop_Codon (100-nucleotide window centered on the stop codon) and 3′UTRs (Duan et al., 2017). We used a python script to assert subtypes of those RNA molecules contain hypermethylated m^6^A peak (scripts can be found in Github^1^). Candidate gene list was submitted to DAVID^2^ to perform GO ontology (GO) and KEGG pathway enrichment analyses.

### Analysis of RNA-seq Data

The input data of m^6^A sequencing was used for transcriptome data analysis. Sequencing reads were trimmed and mapped to the reference genome (TAIR10) by Cutadapt (v1.18) and HISAT2 (v2.1.0), respectively. The differential expression genes (DEG) were identified by R package (DEseq2) (Love et al., 2014) with a cutoff criterion of fold change ≥1.5 and FDR <0.05. Gene ontology (GO) enrichment analyses were performed by using DAVID.

## Results

### *ALKBH10B* Expression is Induced by ABA, JA, Osmotic, and Salt Stress

**FIGURE 1:**
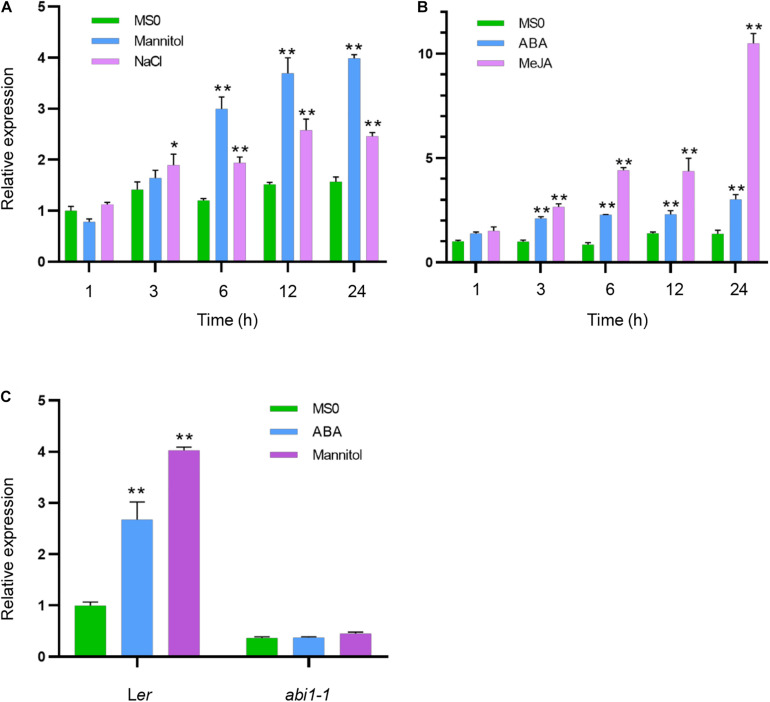
The expression of ALKBH10B is induced by virous external stimuli. **(A,B)** *ALKBH10B* expression in Col-0 plants in response to osmotic stress, salt stress, ABA, and MeJA. Seven-day-old seedlings were transferred to solid 1/2 MS medium, medium with 300 mM mannitol or 150 mM NaCl, or liquid 1/2 MS medium, medium with 50 μM ABA or 50 μM MeJA, respectively. After treated with the indicated time, seedlings were harvested followed by RNA extraction and cDNA synthesis for qRT-PCR. Asterisks indicate statistically significant differences as compared to the mock of every time-point (*P < 0.05; **P < 0.01; Student’s *t*-test). **(C)** *ALKBH10B* expression in *abi1-1* mutant in response to ABA and mannitol. Seven-day-old seedlings were transferred to solid 1/2 MS medium or medium containing 50 μM ABA or 300 mM mannitol for 24 h, respectively. Then seedlings were harvested followed by qRT-PCR. The transcript level of *ALKBH10B* were normalized to the *At2G28390* expression. Error bars represent the standard deviation of three replicates. Asterisks indicate statistically significant differences (*P < 0.05; **P < 0.01; Student’s *t*-test).

In our previous study, we have identified ALKBH10B as an mRNA m^6^A demethylase, which regulates floral transition in Arabidopsis (Duan et al., 2017). In addition to developmental function of ALKBH10B, we asked whether ALKBH10B is involved in abiotic stress response. We firstly examined whether the expression level of *ALKBH10B* is affected by osmotic and salt stress. Seven-day-old seedlings growing on 1/2 MS medium were transferred to solid medium containing 300 mM mannitol or 150 mM NaCl. Seedlings were collected after time-course treatment (1, 3, 6, 12, or 24 h). After treatment with mannitol for 6 h, or NaCl for 3 h, the expression of *ALKBH10B* was significantly increased than that of the control (Figure 1A). This is consistent with the data in AtGenExpress global stress expression database (Kilian et al., 2007). Both ABA and jasmonic acid (JA) are important phytohormones which regulate environment response (Ju et al., 2019). Next, we tested whether *ALKBH10B* expression is activated by ABA and methyl jasmonate (MeJA) treatment. The results showed that after treatment with ABA or MeJA for 3 h, the expression of *ALKBH10B* was significantly increased (Figure 1B).

In ABA signaling pathway, ABA is perceived by the intracellular pyrabactin resistance 1 (PYR1) and PYR1-like (PYL)/regulatory component of ABA receptor (RCAR) proteins. From the transcriptome data of previous studies (Zhao et al., 2018), in *pyl* duodecuple mutant, *pyr1pyl1/2/3/4/5/7/8/9/10/11/12*, the induction of *ALKBH10B* expression by ABA or mannitol was much less in the mutant (Supplementary Figure 1). ABA insensitive 1 (ABI1), encodes group A protein phosphatase 2Cs (PP2C), which regarded as ABA co-receptor (Leung et al., 1994), is crucial for ABA signaling. The activation of *ALKBH10B* expression by ABA or mannitol was blocked in the *abi1-1* mutant (Figure 1C). These results indicated that the expression of *ALKBH10B* is regulated by the PYR/PYL/RCAR-ABI1 mediated ABA signaling pathway.

### *ALKBH10B* Modulates Seed Germination and Post-germination Development Responding to ABA, Salt, and Osmotic Stress

**FIGURE 2:**
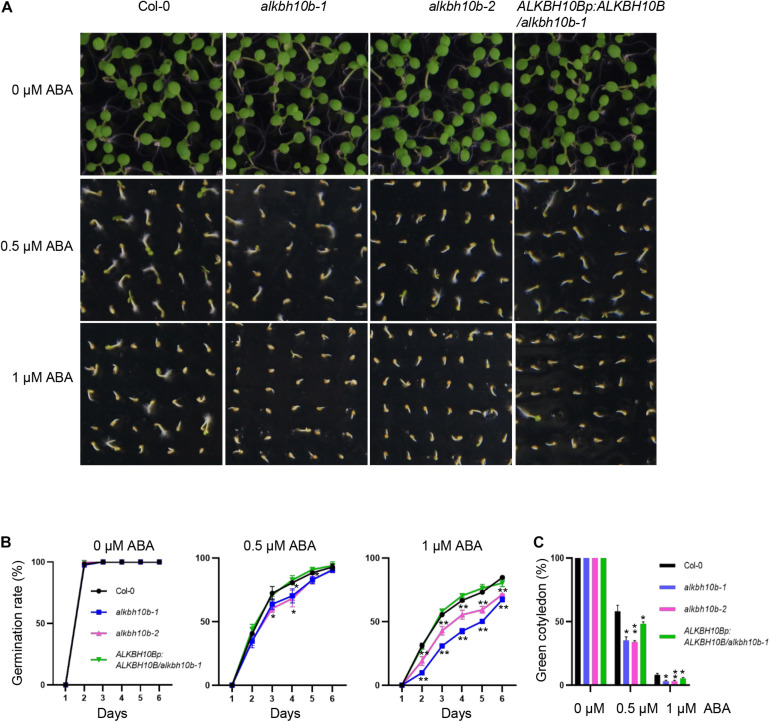
ALKBH10B modulates ABA sensitivity during seed germination. The germination rates of indicated genotypes were recorded from 1–6 days after stratification in the presence of various ABA contents (0, 0.5, or 1.0 μM). Cotyledon-greening percentages of the 10th day were recorded. Three independent experiments were conducted, with at least 100 seeds per genotype in each replicate. Error bars represent the standard deviation of three replicates. Asterisks indicate statistically significant differences as compared to the mock of every time-point (*P < 0.05; **P < 0.01; Student’s *t*-test). **(A)** Photographs of seedings grown on different media at day 6 after stratification. **(B)** Seed germination rates of indicated genotypes grown on different medium. **(C)** Green cotyledon ratio at day 10 after stratification.

The expression of *ALKBH10B* is activated by ABA, suggesting that ALKBH10B plays potential roles in ABA response in Arabidopsis. To understand the roles of ALKBH10B in ABA response, two T-DNA insertion mutants: SALK_004215 (*alkbh10b-1*) and SALK_107289 (*alkbh10b-2*), as well as one complementation material (*ALKBH10Bp:ALKBH10B/alkbh10b-1*) arrived from our previous study were used in the following research. Seed germination is sensitive to ABA concentration; Therefore, we performed the seed germination assay of each genotype under ABA treatment. We germinated the seeds of *alkbh10b-1*, *alkbh10b-2*, *ALKBH10Bp:ALKBH10B/alkbh10b-1*, and wild type (Col-0) on 1/2 MS medium with or without ABA treatment. In the absence of ABA, the germination rate of different genotypes was similar (Figures 2A,B). In the presence of different concentrations of ABA, *alkbh10b-1* and *alkbh10b-2* seeds were more sensitive to ABA inhibition, both mutants showed lower germination rate compared to the wild type (Figures 2A,B). Complementation of ALKBH10B in *alkbh10b-1* (*ALKBH10Bp:ALKBH10B/alkbh10b-1*) recovered the lower germination rate of *alkbh10b-1* under ABA treatment (Figures 2A,B). In line with the germination rate data, *alkbh10b-1* and *alkbh10b-2* mutants also showed lower cotyledon greening rates after 10 days of germination (Figure 2C). These results indicated that ALKBH10B acts as a negative regulator of ABA signaling during seed germination and post-germination growth.

**FIGURE 3:**
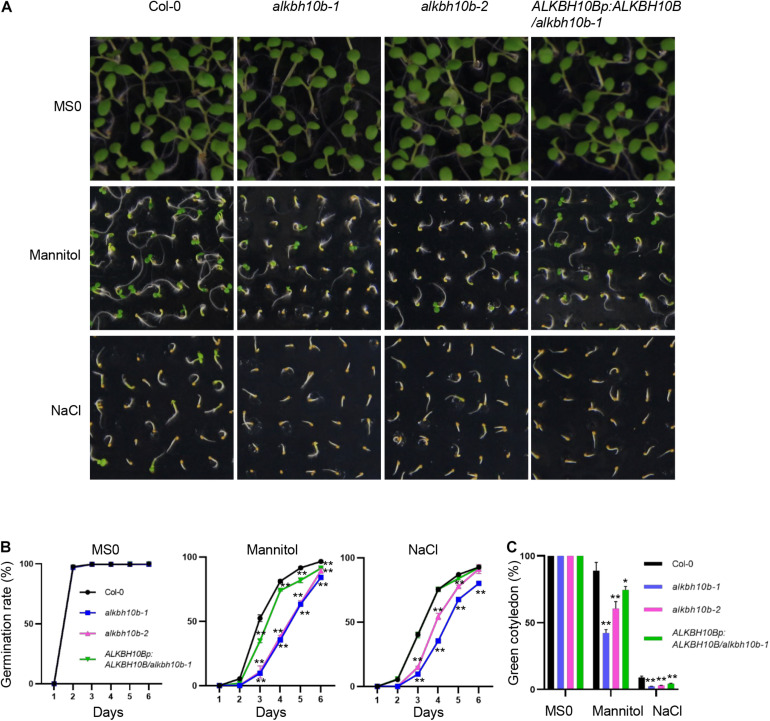
ALKBH10B modulates osmotic and salt stress response during seed germination. The germination rates of indicated genotypes were recorded from 1–6 days after stratification in the presence of 300 mM mannitol or 150 mM NaCl. Cotyledon-greening percentages of the 8th day were recorded. Three independent experiments were conducted, with at least 100 seeds per genotype in each replicate. Error bars represent the standard deviation of three replicates. Asterisks indicate statistically significant differences as compared to the mock of every time-point (*P < 0.05; **P < 0.01; Student’s *t*-test). **(A)** Photographs of seedings grown on different media at day 6 after stratification. **(B)** Seed germination rates of indicated genotypes grown on different medium. **(C)** Green cotyledon ratio at day 8 after stratification.

We also tested whether ALKBH10B affects the seed germination in the presence of osmotic and salt stress. The seeds were sown on 1/2 MS medium supplemented with 300 mM mannitol or 150 mM NaCl. Compared with the wild type seeds, germination and cotyledon greening ratios of *alkbh10b-1* and *alkbh10b-2* seeds were more inhibited under mannitol or NaCl treatment (Figures 3A–C), indicating that ALKBH10B plays roles in regulating seed germination and post-germination growth in response to osmotic and salt stress.

### Transcriptome Analysis of *alkbh10b-1* After ABA Treatment

**FIGURE 4:**
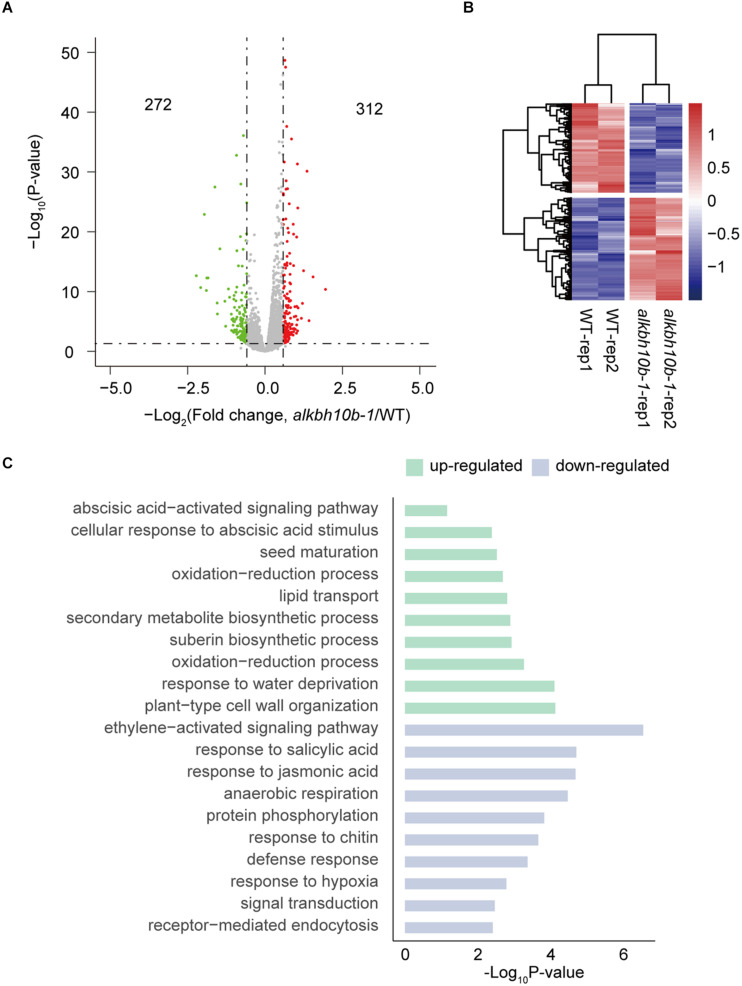
Differential expression genes identified in *alkbh10b-1* compared with Col-0. **(A)** Volcano plots showing the differentially expressed genes identified in *alkbh10b-1*. **(B)** Heatmap plots showing the normalized RPKM of differentially expressed genes in *alkbh10b-1*. **(C)** Biological process enrichment analysis of the upregulated and downregulated genes in *alkbh10b-1*.

Since both *alkbh10b* mutants are more sensitive to ABA than Col-0, we speculated that ALKBH10B may affects the expression of some ABA response genes. Therefore, we performed RNA sequencing experiments to profile the transcriptome of *alkbh10b-1* and the wild type after ABA treatment. Total RNA was isolated from 7-day-old seedlings treated with ABA for 3 h and used for RNA-seq library construction. We identified 312 upregulated genes and 272 downregulated genes (fold change ≥1.5 and FDR ≤0.05) (Figure 4A and Supplementary Dataset 2). Hierarchical clustering depicted differential expression profiles in all samples (Figure 4B). Then we performed GO analysis of these DEG using DAVID. The results showed that the upregulated genes were significantly enriched in cellular response to ABA stimulus, ABA activated signaling pathway, seed maturation and water deprivation (Figure 4C), which suggest that ALKBH10B may participates in ABA signaling pathway by affecting genes involved in ABA signaling pathway and plays a role in seed germination, which corresponds to the phenotype of *alkbh10b* mutation lines. Furthermore, downregulated genes were significantly enriched in the response of plant hormone signaling, including salicylic acid and JA (Figure 4C), which are import in various stress response and signal transduction. These results suggesting ALKBH10B plays a role in abiotic stress response and system defense.

### ALKBH10B Negatively Affects the Expression of ABA Response Genes

**FIGURE 5:**
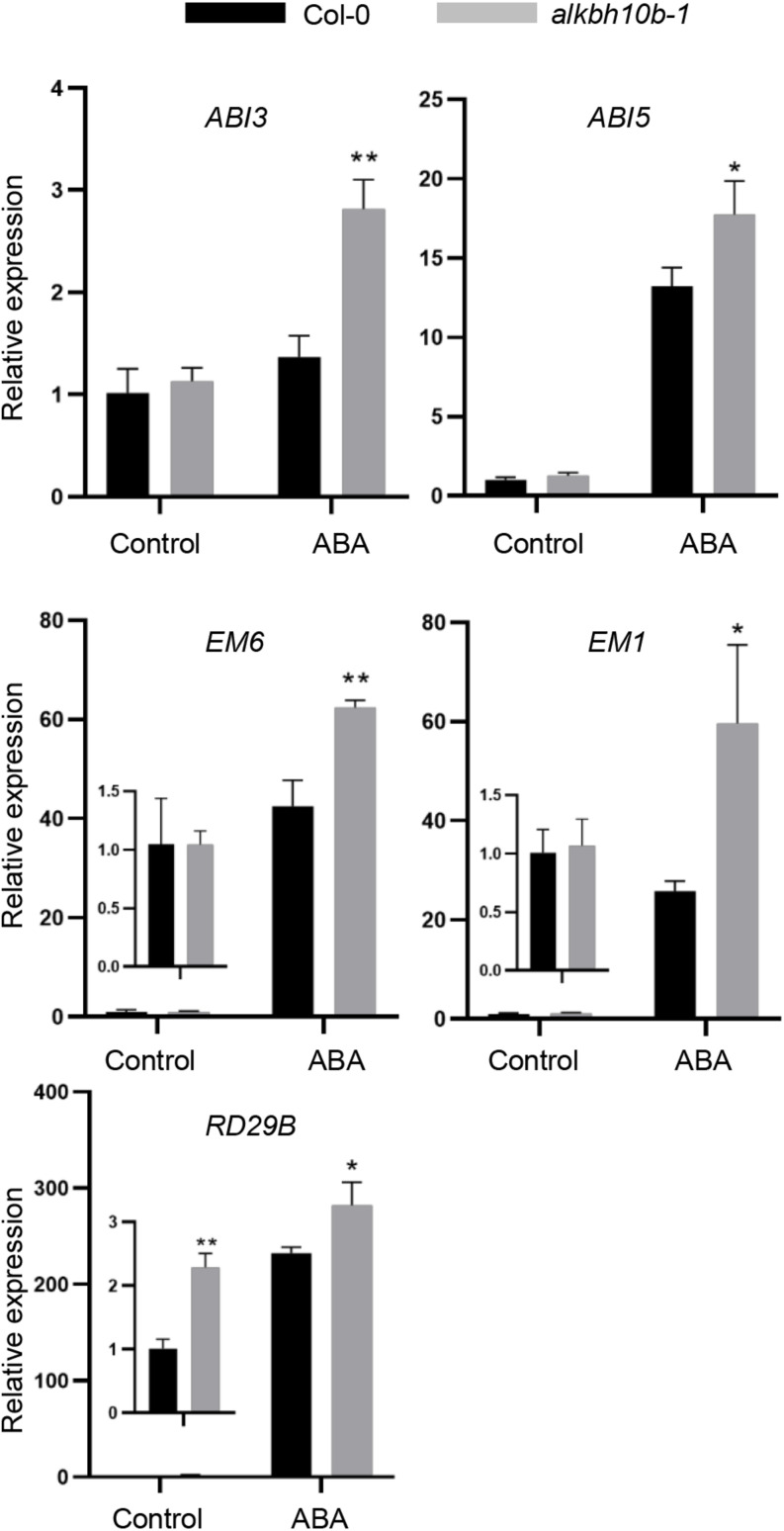
ALKBH10B regulates the expression of ABA response genes. The expression patterns of ABA signaling-related ABI3, ABI5, and RD29B, and embryogenesis-related EM1 and EM6 were determined in seven-day-old seedlings treated with 50 μM ABA for 3 h. The transcript level of each gene was normalized to that of *At2G28390*, and the relative level of each gene in mock-treated Col-0 was set to 1. Values are mean ± SD of three replications. Asterisks indicate statistically significant differences (*P < 0.05; **P < 0.01; Student’s *t*-test).

RNA-seq data revealed that the upregulated genes were significantly enriched in ABA response and seed maturation, which may explain why the *alkbh10b* mutants were more sensitive under ABA treatment. ABI3 and ABI5 are two important transcription factors in regulating seed maturation, germination, and early development of seedlings (Bensmihen et al., 2002; Lopez-Molina et al., 2002). We measured the transcript levels of *ABI3*, *ABI5*, and some of their downstream genes, including *RD29B*, *EM6*, and *EM1* in Col-0 and *alkbh10-1* using qRT-PCR under mock and ABA treatment. The results showed that the expression of *ABI3*, *ABI5*, *EM6*, *EM1*, and *RD29B* were upregulated in *alkbh10b-1* after ABA treatment (Figure 5). Among them, the higher expression of *EM6* and *RD29B* in *alkbh10b-1* was consistent with the RNA-seq data; the higher expression of *ABI3*, *ABI5*, and *EM1* were not identified in the RNA-Seq data because of their low expression or their insignificant difference between *alkbh10b-1* and wild type. To better understand the role of ALKBH10B in seed germination, we detected the expression patterns of these ABA signaling-related genes in seeds germinated for 2.5 days on medium containing 0 or 0.5 μM ABA. The results showed that the expression of *ABI3*, *ABI5*, *EM6*, *EM1*, and *RD29B* in *alkbh10b-1* was higher than that of the Col-0 on medium with or without ABA (Supplementary Figure 2). Above all, our results showed that ALKBH10B negatively affects the expression of ABA response genes.

### Transcriptome-Wide Analysis of mRNA m^6^A Modification in *alkbh10b-1* After ABA Treatment

**FIGURE 6:**
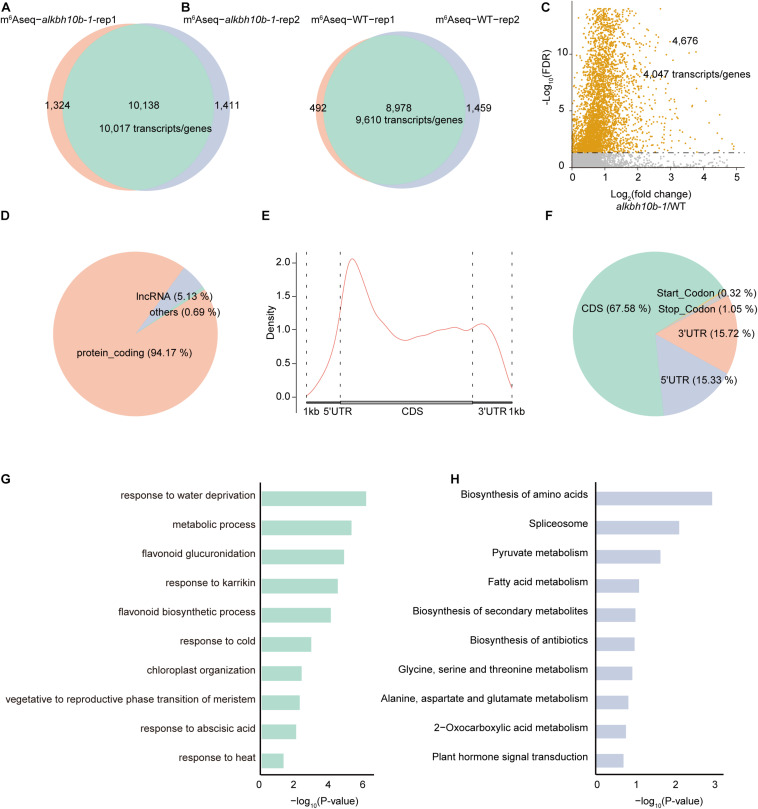
Characterization of m^6^A modification in Col-0 and *alkbh10b-1* under ABA treatment. **(A)** Overlap of two biological replicates of m^6^A enriched peaks in *alkbh10b-1*. **(B)** Overlap of two biological replicates of m^6^A enriched peaks in Col-0. **(C)** Volcano plot showing the hypermethylated m^6^A peaks identified in *alkbh10b-1* compared with Col-0. **(D)** Pie chart depicting RNA types of hypermethylated m^6^A peaks in *alkbh10b-1*. **(E)** Metagene profile showing the density of hypermethylated m^6^A peaks across the transcript. **(F)** Pie chart depicting the fraction of the hypermethylated m^6^A peaks in the five non-overlapping transcript segments [5′UTR, start codon, coding sequence (CDS), stop codon and 3′UTR]. **(G)** Biological process enrichment analysis of genes with hypermethylated m^6^A peaks. **(H)** Pathway analysis of genes with hypermethylated m^6^A peaks.

**FIGURE 7:**
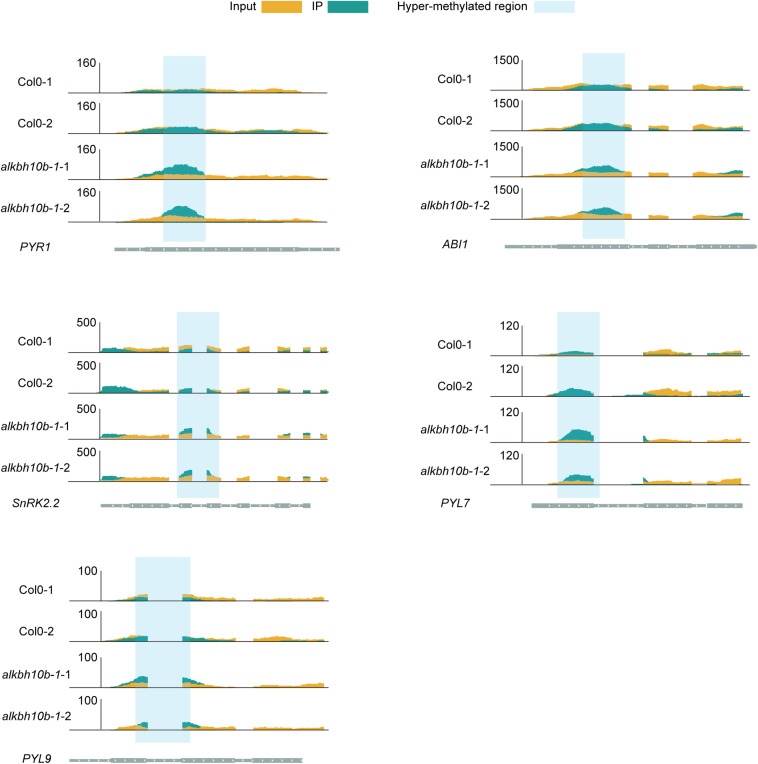
Integrative genomics viewer (IGV) tracks showing the hypermethylated m^6^A peaks of several ABA signaling genes identified in *alkbh10b-1*.

To investigate whether m^6^A methylation pattern is different in *alkbh10b* mutant compared to wild type under 50 μM ABA treatment, we performed m^6^A-seq in *alkbh10b-1* and Col-0, respectively. After removing PCR duplication, approximately 10.0–21.6 million reads were mapped to TAIR10 genome with a more than 90% mapping rate (Supplementary Dataset 1). We used MACS2, a well-known peak caller, to identify peaks with a criteria of fold enrichment (IP/input) ≥2 and FDR ≤0.05. The m^6^A peaks identified in both replicates were used for further analysis. We identified 10,138 m^6^A peaks corresponding to 10,017 transcripts/genes in *alkbh10b-1* mutant (Figure 6A), whereas 8,978 m^6^A peaks corresponding to 9,610 transcripts/genes in Col-0 (Figure 6B), indicating that loss of function of ALKBH10B caused an increase of m^6^A peak numbers. We subsequently identified hypermethylated m^6^A peaks in *alkbh10b-1* mutant under ABA treatment using the MeTDiff R package software (FDR ≤ 0.05). Compared with WT, a total of 4,676 peaks were identified in *alkbh10b-1*, corresponding to 4,047 transcripts/genes (Figure 6C and Supplementary Dataset 3), which were mainly mRNA (94.17%), only ∼5.13% were lncRNA and a small proportion were other RNAs (Figure 6D). Then we examined the distribution profiles of these hypermethylated m^6^A peaks within the transcripts, and found that the density of these peaks tends to be enriched in CDS, near the start codon and 5′UTR (Figure 6E), which is consistent with the distribution of hypermethylated peaks in normal condition (Duan et al., 2017). To further locate these m^6^A peaks, we assigned these m^6^A peaks into five-non-overlapping regions and found that these hypermethylated peaks were dominantly enriched in CDS (67.58%), then 3′UTR (15.72%) and 5′UTR (15.33%) (Figure 6F). To explore the function of the hypermethylated m^6^A genes in *alkbh10b-1* mutant under ABA treatment, we performed GO enrichment analysis using DAVID. The results revealed that genes with hypermethylated m^6^A peaks are enriched in various abiotic biological processes including response to water deprivation, cold, ABA, and heat (Figure 6G). KEGG pathway analysis showed that genes with hypermethylated m^6^A peaks are mainly enriched in biosynthesis of amino acids, pyruvate metabolism and plant hormone signal transduction (Figure 6H). In addition, several ABA signaling genes, including *PYR1*, *PYL7*, *PYL9*, *ABI1*, and *SnRK2.2* were m^6^A hypermethylated in *alkbh10b-1* under ABA treatment compared with WT (Figure 7). We also analyzed the m^6^A-seq data (Duan et al., 2017) between *alkbh10b-1* and Col-0 under normal conditions. None of these genes were m^6^A hypermethylated in *alkbh10b-1* under normal conditions (Supplementary Figure 3), suggesting m^6^A hypermethylation of these genes may specific to ABA treatment. These results indicated that ALKBH10B may regulates ABA response by affecting the m^6^A level of these ABA signaling genes.

## Discussion

More and more studies have reported the importance of mRNA m^6^A modification in plant development, including embryo development (Zhong et al., 2008; Shen et al., 2016; Ruzicka et al., 2017), microspore development (Zhang et al., 2019), shoot apical meristem proliferation (Shen et al., 2016), trichome morphology (Arribas-Hernandez et al., 2018; Scutenaire et al., 2018; Wei et al., 2018), floral transition (Duan et al., 2017), and fruit ripping (Zhou et al., 2019). mRNA m^6^A modification also plays roles in biotic and abiotic stress response, for example, virus infection (Martinez-Perez et al., 2017), and salt stress response (Hu et al., 2021). Plant seeds need to identify and respond to different environmental factors to decide whether to germinate or not. Even after seed germination, the seedlings will feel the changes of the surrounding environment and inhibit growth in a process called post-germination developmental arrest. Abscisic acid (ABA) is an important hormone to balance early growth or inhibition. Here, we found that ALKBH10B, an mRNA m^6^A demethylase, modulates seed germination by regulating ABA response genes.

We demonstrated that ALKBH10B plays a role in seed germination through an ABA dependent manner based on the following evidence. First, the expression of *ALKBH10B* is induced by ABA, NaCl, and mannitol stress, however, in *abi1-1*, the inducibility of *ALKBH10B* is disappeared (Figure 1 and Supplementary Figure 1), suggesting that *ALKBH10B* is regulated by ABA signaling and work downstream of ABI1. Second, the seeds of *alkbh10b* mutants were more sensitive than that of the wild type in response to ABA, osmotic, and salt stress, and showed lower germination rate and cotyledons greening at the same day (Figures 2, 3). Third, RNA-seq data reveals that, ABA response genes were upregulated in *alkbh10b-1* after ABA treatment. We found that transcription factors, *ABI3* and *ABI5*, which are two well-known positive regulators of ABA signaling during seed germination, were upregulated in *alkbh10b-1* after ABA treatment (Figures 4, 5). Fourth, some ABA response genes were m^6^A hypermethylated in *alkbh10-1*, including *PYR1*, *PYL4*, *PYL7*, *PYL9*, and *ABI1*, suggesting the role of ALKBH10B-mediated m^6^A demethylation in ABA signaling gene regulation. Taken together, our results indicate that ALKBH10B modulated seed germination by negatively affecting ABA response genes.

The mechanism of m^6^A-mediated gene regulated is complicated. So far, researchers have found virous mechanism in mammals, including RNA stability maintenance, alternative splicing, mRNA nuclear export, translation regulation, and pri-miRNA processing (Fustin et al., 2013; Wang et al., 2014, 2015; Alarcon et al., 2015; Meyer et al., 2015; Xiao et al., 2016). Recently, some reports found that RNA m^6^A modification can modulate chromatin accessibility in mammalian cells, mainly through its “writer” or “reader” proteins to recruit histone modification proteins to change the histone modification state (Li et al., 2020; Liu et al., 2020, 2021; Xu et al., 2021), increasing the complexity of m^6^A regulation mechanism. Although, we found ALKBH10B negatively regulates the expression of ABA signaling genes under ABA treatment, and found some ABA response genes were m^6^A hypermethylated, the specific mechanism of how ALKBH10B modulates the expression of ABA response genes needs further study.

## Conclusion

Here, we found that the expression of *ALKBH10B* is induced in response to ABA, osmotic, and salt stress, which depends on ABI1. The *alkbh10b* mutants showed more sensitive to ABA, osmotic, and salt stress during seed germination than the wild type. The expression of several ABA response genes was upregulated in *alkbh10b-1* than that of wild type, indicating ALKBH10B negatively affects the ABA signaling. Furthermore, mRNA m^6^A sequencing found that mRNA of ABA signaling genes, including *PYR1*, *ABI1*, *PYL7*, *PYL9*, and *SnRK2.2* were m^6^A hypermethylated in *alkbh10b-1* after ABA treatment. However, further studies are needed to explain the specific mechanism of how ALKBH10B modulating the expression of ABA response genes.

## Data Availability Statement

The datasets presented in this study can be found in online repositories. The names of the repository/repositories and accession number(s) can be found below: https://bigd.big.ac.cn/gsa/s/uPCjOJ90, PRJCA005164.

## Author Contributions

JT performed the experiments with the help of HD. JY analyzed the sequencing data. GJ and JT designed the experiments, interpreted the results, and wrote the manuscript. All authors contributed to the article and approved the submitted version.

## Conflict of Interest

The authors declare that the research was conducted in the absence of any commercial or financial relationships that could be construed as a potential conflict of interest.

## Publisher’s Note

All claims expressed in this article are solely those of the authors and do not necessarily represent those of their affiliated organizations, or those of the publisher, the editors and the reviewers. Any product that may be evaluated in this article, or claim that may be made by its manufacturer, is not guaranteed or endorsed by the publisher.

## Footnotes

1
https://github.com/joybio/m${}^{6}$A-seq/tree/main/feature_annotation/

2
https://david.ncifcrf.gov/tools.jsp
