# Supplementary material for: ALKBH10B, an mRNA m6A Demethylase, Modulates ABA Response During Seed Germination in Arabidopsis
Source: Front Plant Sci. 2021 Jul 27;12:712713. doi: 10.3389/fpls.2021.712713 (PMC8353332; doi:10.3389/fpls.2021.712713)
Supplement: Supplementary file 1 [file Data_Sheet_1.PDF]

Supplementary Figures

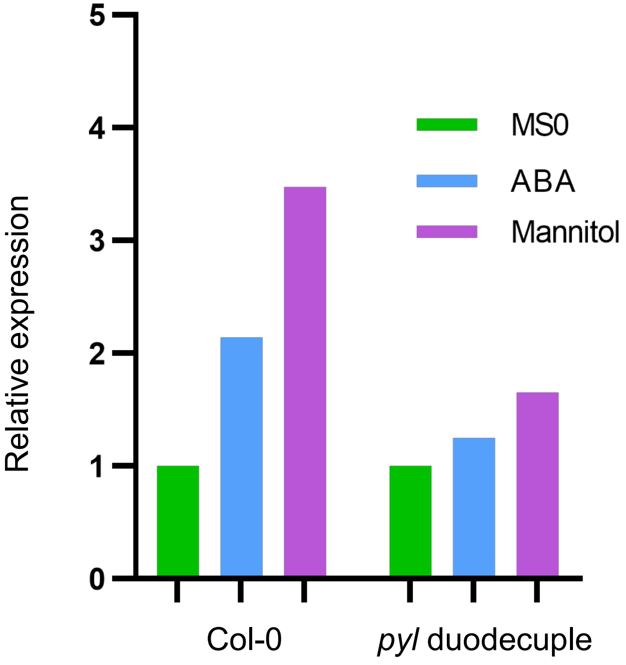

Figure S1. *ALKBH10B* expression in *pyl* duodecuple mutant in response to ABA and mannitol. Data from the transcriptome results of previous research (Zhao et al., 2018).

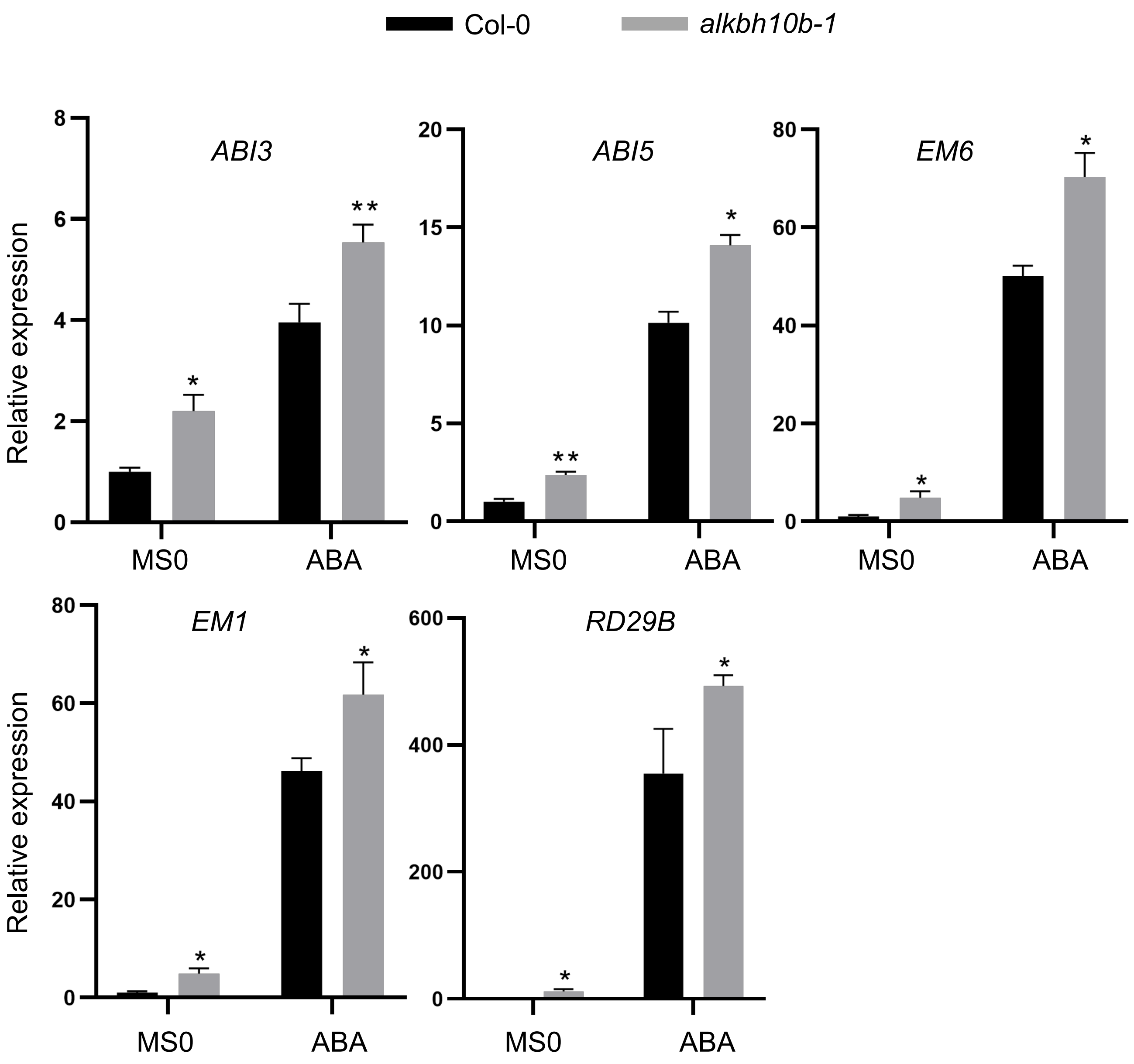

Figure S2. ALKBH10B regulates the expression of ABA response genes in germinating seeds. The expression patterns of ABA signaling-related ABI3, ABI5, and RD29B, and embryogenesis-related EM1 and EM6 were determined in seeds germinated for 2.5 d on medium containing 0 or 0.5 μM ABA. The transcript level of each gene was normalized to that of *At2G28390*, and the relative level of each gene in mock-treated Col-0 was set to 1. Values are mean ± SD of three replications. Asterisks indicate statistically significant differences (*P < 0.05 and **P < 0.01; Student's t test).

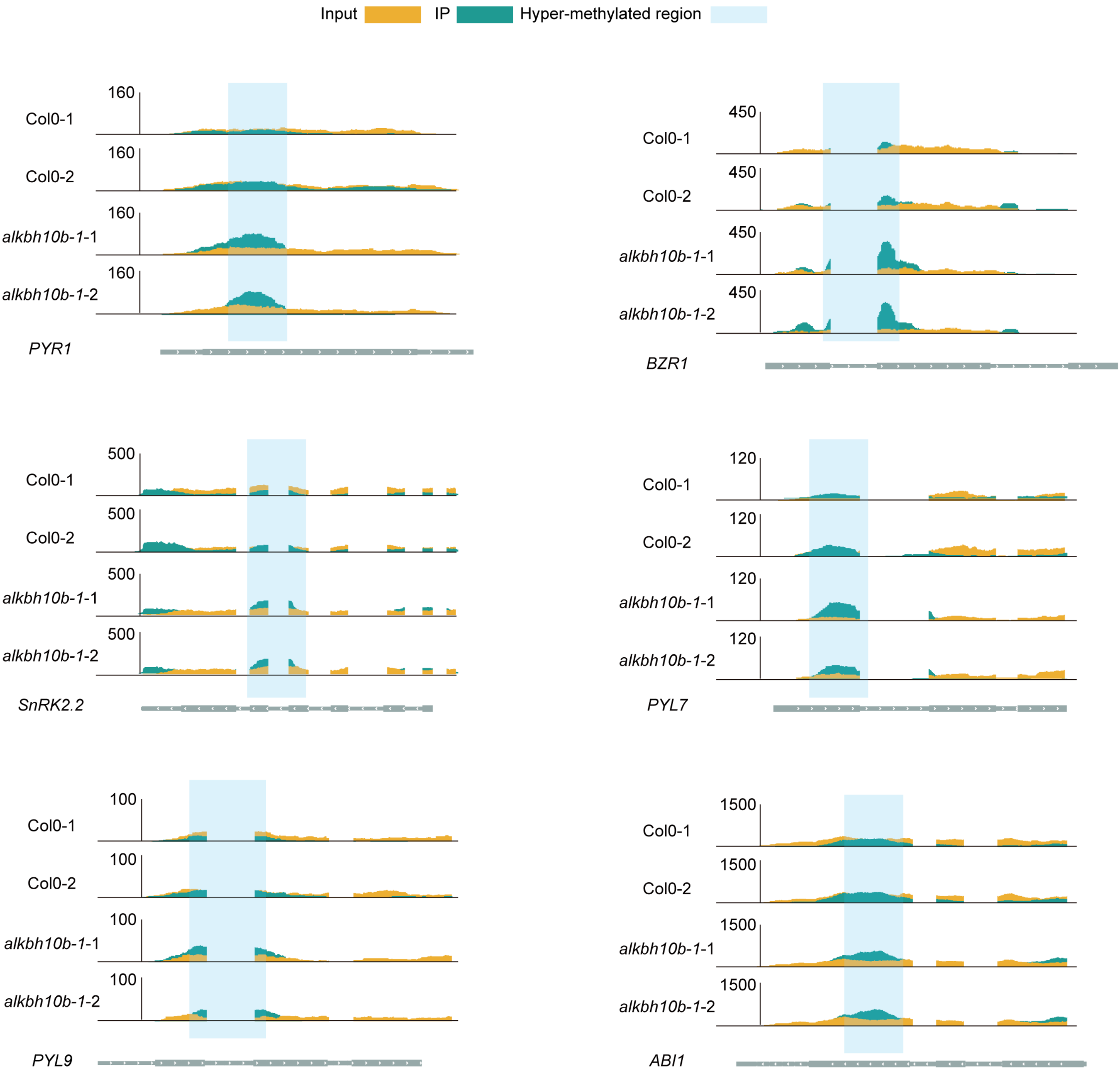

Figure S3. Integrative genomics viewer (IGV) tracks of *ABI1*, *PYL7*, *PYL9*, *PYR1*, and *SnRK2.2* in m^{6}A-seq results from Duan et al., 2017 between *alkbh10b-1* and Col-0 under normal conditions.
